# Unfoldomics of human diseases: linking protein intrinsic disorder with diseases

**DOI:** 10.1186/1471-2164-10-S1-S7

**Published:** 2009-07-07

**Authors:** Vladimir N Uversky, Christopher J Oldfield, Uros Midic, Hongbo Xie, Bin Xue, Slobodan Vucetic, Lilia M Iakoucheva, Zoran Obradovic, A Keith Dunker

**Affiliations:** 1 Center for Computational Biology and Bioinformatics, Indiana University School of Medicine, Indianapolis, IN 46202, USA; 2 Institute for Intrinsically Disordered Protein Research, Indiana University School of Medicine, Indianapolis, IN 46202, USA; 3 Institute for Biological Instrumentation, Russian Academy of Sciences, 142290 Pushchino, Moscow Region, Russia; 4 Center for Information Science and Technology, Temple University, Philadelphia, PA 19122, USA; 5 Laboratory of Statistical Genetics, The Rockefeller University, New York, NY 10065 USA

## Abstract

**Background:**

Intrinsically disordered proteins (IDPs) and intrinsically disordered regions (IDRs) lack stable tertiary and/or secondary structure yet fulfills key biological functions. The recent recognition of IDPs and IDRs is leading to an entire field aimed at their systematic structural characterization and at determination of their mechanisms of action. Bioinformatics studies showed that IDPs and IDRs are highly abundant in different proteomes and carry out mostly regulatory functions related to molecular recognition and signal transduction. These activities complement the functions of structured proteins. IDPs and IDRs were shown to participate in both one-to-many and many-to-one signaling. Alternative splicing and posttranslational modifications are frequently used to tune the IDP functionality. Several individual IDPs were shown to be associated with human diseases, such as cancer, cardiovascular disease, amyloidoses, diabetes, neurodegenerative diseases, and others. This raises questions regarding the involvement of IDPs and IDRs in various diseases.

**Results:**

IDPs and IDRs were shown to be highly abundant in proteins associated with various human maladies. As the number of IDPs related to various diseases was found to be very large, the concepts of the disease-related unfoldome and unfoldomics were introduced. Novel bioinformatics tools were proposed to populate and characterize the disease-associated unfoldome. Structural characterization of the members of the disease-related unfoldome requires specialized experimental approaches. IDPs possess a number of unique structural and functional features that determine their broad involvement into the pathogenesis of various diseases.

**Conclusion:**

Proteins associated with various human diseases are enriched in intrinsic disorder. These disease-associated IDPs and IDRs are real, abundant, diversified, vital, and dynamic. These proteins and regions comprise the disease-related unfoldome, which covers a significant part of the human proteome. Profound association between intrinsic disorder and various human diseases is determined by a set of unique structural and functional characteristics of IDPs and IDRs. Unfoldomics of human diseases utilizes unrivaled bioinformatics and experimental techniques, paves the road for better understanding of human diseases, their pathogenesis and molecular mechanisms, and helps develop new strategies for the analysis of disease-related proteins.

## Background

### Introducing intrinsically disordered proteins

Proteins are the major components of the living cell. They play crucial roles in the maintenance of life. Protein dysfunctions may cause development of various pathological conditions For more than 75 years it has been believed that the specific functionality of a given protein is predetermined by its unique 3-D structure [1,2]. For these structured proteins, the sequence → structure → function paradigm has become paramount. According to this view, a protein's function depends on its prior folding into a unique three-dimensional structure. In such cases, the amino acid sequence determines the protein's unique 3-D structure.

Evidence is rapidly accumulating that many protein regions and even entire proteins lack stable tertiary and/or secondary structure in solution yet possess crucial biological functions [3-25]. These naturally flexible proteins and regions are known by different names, including intrinsically disordered [8], natively denatured [26], natively unfolded [27], intrinsically unstructured [4], natively disordered [21], and inherently disordered [25,28,29]. In this article, the terms "intrinsically disordered proteins" and "intrinsically disordered regions" (IDPs and IDRs, respectively) are used to describe such proteins and regions generally and "natively denatured" or "intrinsically unstructured" are used for collapsed and extended random coils that lack significant amounts of stable secondary structure (see below).

IDPs and IDRs can contain collapsed-disorder, semi-collapsed disorder, or extended-disorder under physiological conditions *in vitro *[6,12,19]. Collapsed-disorder consists mainly of molten globules, which are formed by hydrophobic collapse, which have stable but dynamic secondary structure, and which have flexible and dynamic side chains [14,30-38]. Semi-collapsed structures arise because water is a poor solvent for the peptide backbone and include, for example, polyglutamine regions [28], other polar sequences [29], and pre-molten globules [14,39-41]. Pre-molten globules may contain regions with transient secondary structure or small amounts of localized, fairly stable secondary structure. Extended-disorder arises from chains having repulsion arising from a net charge, and these proteins and regions resemble the more classical idealized random coil. Because of the lack of a hydrophobic core and the presence of only the marginal levels of residual secondary structure, native coils and native pre-molten globules are grouped together in a class of natively unfolded or intrinsically unstructured proteins [11,12].

**Figure 1:**
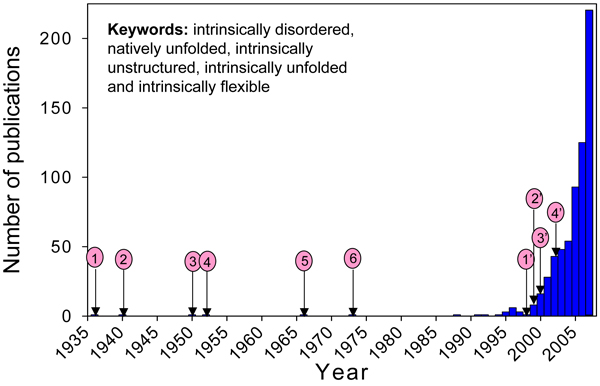
**Increase in the number of publications dealing with the IDPs**. Circles 1–6 correspond to some key IDP-related publications in the pre-bioinformatics era. They are: 1) Landsteiner, 1936 [110]; 2) Pauling, 1940 [111]; 3) Karush, 1950 [112]; 4) McMeekin, 1962 [113]; 5) Jirgenesons, 1966 [114]; 6) Doolittle, 1973 [115]. Circles 1'-4' correspond to key research bioinformatics articles and reviews that created and shaped the IDP field: 1') Romero et al., 1998 [3]; 2') Wright & Dyson, 1999 [4]; 3') Uversky et al., 2000 [6]; 4') Tompa, 2002 [13].

In contrast to the long history regarding structured proteins, the study of the IDP phenomenon is emerging only very recently (Figure 1). This transition is occurring mostly due to the efforts of four research groups, which almost simultaneously and completely independently came to the important conclusion that naturally flexible proteins, instead of being just rare exceptions, represent a new and very broad class of proteins [1,2,4,11]. This important conclusion was reached from different starting points using very different experimental approaches, including: bioinformatics (Dr. A.K. Dunker's group), NMR spectroscopy (Dr. P.E. Wright's group), multiparametric protein folding/misfolding studies (Dr. V.N. Uversky's group), and protein structural characterization (Dr. P. Tompa's group). The bioinformatics approach has played an especially crucial role in shaping this field, bringing coherence and recognition to proteins that were previously viewed individually as outliers from the main stream [42]. After publication of key studies and reviews describing this new concept, the literature on IDPs and IDRs is virtually exploding (see Figure 1).

**Figure 2:**
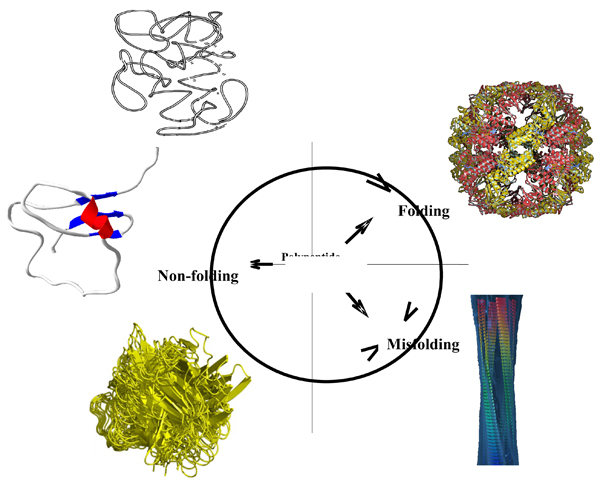
**The modern understanding of the fate of a polypeptide chain inside a cell**. Three types of IDPs, native coil, native pre-molten globule, and native molten globule are schematically shown together with the structure of an ordered protein and an amyloid fibril. Here, newly synthesized polypeptide chain can either undergo specific folding to gain a unique structure necessary for the catalytic and transport activities, or stay substantially non-folded or misfold and form amyloid-like fibrils. Both folded and non-folded proteins have specific biological functions.

Figure 2 represents the modern understanding of the fate of a polypeptide chain inside a cell and schematically shows the three types of intrinsic disorder mentioned above, native coil, native pre-molten globule, and native molten globule. According to this hypothesis, newly synthesized proteins can either fold to gain a unique structure necessary for the catalytic and transport activities, can stay substantially non-folded, or can misfold under some circumstances to form amyloid-like fibrils. Importantly, both folded and non-folded polypeptide chains have specific biological functions. The three endpoints are further interlinked and some changes in the environment, interaction with specific binding partners, or mutations may bring subsequent structural rearrangements. As a result, an intrinsically disordered polypeptide can partially or completely fold or misfold and form amyloid-like fibrils, whereas ordered protein can likewise misfold and assemble into a pathogenic fibrillar form (see Figure 2) [14].

Just as the amino acid sequence of an ordered protein contains the information for a highly specific folding, the amino acid sequence of an IDP codes for lack of structure or disorder. The validity of this hypothesis is supported by the development of various disorder predictors [29-32] all based on well-defined biases in the IDP sequences and amino acid compositions [3,6,7,42-45]. For example, natively unfolded proteins were shown to be specifically localized within a unique region of charge-hydropathy phase space characterized by a combination of low overall hydropathy and high net charge [6]. More specifically, in comparison with structured proteins, IDPs and IDRs are generally depleted in the structure-promoting residues (including cysteine, tryptophan, tyrosine, isoleucine, phenylalanine, valine, leucine, histidine, threonine, and asparagine) and noticeably enriched in the disorder-promoting residues (aspartic acid, methionine, lysine, arginine, serine, glutamine, proline, and glutamic acid) [5,29,33,34].

IDPs and IDRs are highly abundant in nature. This follows from the results of disorder prediction for many whole proteomes. The fraction of proteins with substantial amounts of disorder is found to be proportional to the complexity of the organisms. IDPs/IDRs are more abundant in eukaryotes than in archaea and prokaryotes. Furthermore, multicellular eukaryotes were shown to have much more predicted disorder than unicellular eukaryotes [5,46,47]. In general, for mammals, ~75% of their signaling proteins are predicted to contain long IDRs (> 30 residues), about half of their total proteins are predicted to contain such long IDRs, and ~25% of their proteins are predicted to be fully disordered [25].

IDPs and IDRs carry out pivotal biological functions, participating in recognition and in various signaling and regulatory pathways, via specific protein-protein, protein-nucleic acid and protein-ligand interactions [22,48-50]. Sites of various post-translational modifications (PTMs) and sites of proteolytic attack are frequently associated with regions of intrinsic disorder [50]. The capability of non-folding proteins and regions to interact with collections of partners is utilized in organizing complex protein-protein interaction networks. In fact, hub proteins have been shown to have multiple interactions, either being intrinsically disordered and serving as an anchor, or acting as a stable globular anchor that interacts with intrinsically disordered regions of its targets [21,51-56].

Summarizing, whole proteins or protein regions are intrinsically disordered if they fail to fold into 3-D structures, remaining as floppy ensembles with specific biological functions. In our view, IDPs include molten globules, pre-molten globules, random coils and transiently structured forms. IDPs are highly abundant and carry out numerous vital functions. IDPs and IDRs can be predicted by a variety of algorithms. Experimentally, they can be identified using various biophysical techniques, including NMR (especially ^1^H-^15^N NOEs), X-ray crystallography (especially missing density regions), circular dichroism, protease sensitivity, and many others [57].

## IDPs in human diseases: illustrative case studies

Proteins are involved in the maintenance of all stages of the life cycle. The fact that protein dysfunction can cause development of various pathological conditions was known for a very long time. Currently, a broad range of human diseases is linked to the failure of a specific peptide or protein to adopt its functional conformational state; i.e., to protein misfolding, loss of normal function, gain of toxic function, and/or protein aggregation. Although each of such diseases originates from the misfunction of a particular protein, they all are grouped together as protein-conformation or protein-misfolding diseases to emphasize the common molecular mechanisms of their origin. Triggers for misfolding vary for different proteins. Some disease-related proteins have an intrinsic propensity to form pathologic conformation(s). For other proteins, interactions or impaired interactions with chaperones, intracellular or extracellular matrixes, other proteins, small molecules and other endogenous factors can induce conformational changes and increase propensity to misfold. Often, misfolding and misfunction originate from point mutation(s) or result from an exposure to internal or external toxins, impaired posttranslational modifications (phosphorylation, advanced glycation, deamidation, racemization, etc.), an increased probability of degradation, impaired trafficking, lost binding partners or oxidative damage. All these factors can act independently, additively or synergistically.

Protein-conformation diseases can affect a single organ or be spread through multiple tissues. For example, numerous amyloidoses and various neurodegenerative disorders originate from the conversion of specific proteins from their soluble functional states into stable, highly ordered amyloid fibrils, and from the deposition of these aggregates in the variety of organs and tissues. Although protein aggregation is the most visible and the best studied consequence of protein misfolding, pathogenesis of many human diseases might depend on more subtle structural changes that lead to misfunction. Many of the proteins associated with the various conformational diseases are involved in recognition, regulation and cell signaling and a great number of these proteins are IDPs. This review is an attempt to develop an overall understanding of the roles of IDPs in various human diseases. We will start with a couple of illustrative examples where well-characterized IDPs were shown to be associated with the pathogenesis of specific diseases. We will consider here α-synuclein, p53 and HPV proteins. Additional illustrative examples can be found in our recent review [58]. The abundance of intrinsic disorder in various disease-associated proteins will be revealed using specific bioinformatics and computational tools. Then, we will attempt to answer the question why IDPs are so frequently associated with human diseases. The overall goal of this review is to introduce a concept of the disease-related unfoldome and to describe a set of bioinformatics approaches that serve as specific unfoldomics tools.

### α-Synuclein, Parkinson's Diseases and other synucleinpathies

α-Synuclein is one of the most intensively studied IDPs [59-61]. This is because of its association with a group of neurodegenerative disorders, synucleinopathies, characterized by the fibrillar α-synuclein aggregates in the cytoplasm of selective populations of neurons and glia [62-65] and by a chronic and progressive decline in motor, cognitive, behavioral, and autonomic functions, with the disease phenotype depending on the distribution of the lesions. Some of the most common synucleinopathies are Parkinson's disease (PD), dementia with Lewy bodies (DLB), Alzheimer's disease (AD), Down's syndrome, multiple system atrophy (MSA), and neurodegeneration with brain iron accumulation type 1 (NBIA1). A more complete list of synucleinopathies is shown in Additional file 1.

Depending on the type of pathology, α-synuclein inclusions are present in neurons (both dopaminergic and non-dopaminargic), where they can be deposited in perikarya or in axonal processes of neurons, and in glia. At least five morphologically different α-synuclein-containing inclusions have been described: Lewy bodies, Lewy neurites (dystrophic neurites), glial cytoplasmic inclusions, neuronal cytoplasmic inclusions and axonal spheroids [60,61].

The protein that links various synucleinopathies is α-synuclein, which is a typical IDP with low level of ordered structure under the physiological conditions *in vitro *[59]. According to the detailed conformational studies, the structure of α-synuclein is extremely sensitive to the environment, and this protein is known to adopt a variety of structurally unrelated conformations. The list includes a natively unfolded (mostly disordered) state, an amyloidogenic partially folded conformation, and different α-helical or β-structural species folded to a different degree, both monomeric and oligomeric [59]. It might also form aggregates with different morphology, oligomers (spherical or annular), amorphous aggregates, and amyloid-like fibrils [59]. Finally, similar to other fibrillating proteins [66], α-synuclein was shown to assemble into the annular aggregates able to form ion-conducting, transmembrane channels [67-69]. As α-synuclein has a high intrinsic propensity to aggregate, it represents a unique model for the structural and mechanistic analysis of amyloidogenic IDPs.

To describe the structural malleability of α-synuclein, the concept of a protein-chameleon was proposed, according to which the structure of α-synuclein depends on its environment and the choice between various conformations is determined by the peculiarities of the protein's surroundings [59]. This conformational plasticity is determined by a specific folding-energy landscape of an IDP, which in contrast to that of an ordered protein, is characterized by numerous local energy minima, leading to a highly frustrated system without any stable well-folded conformation [58]. Such an energy landscape can explain the conformational plasticity of an IDP and show how such a protein can specifically interact with many ligands of different nature and respond differently to various environmental challenges. The interaction with a particular binding partner (or other changes in the environment) affects the IDP folding landscape making some energy minima deeper and some energy barriers higher, therefore determining the ability of such a protein to fold in a template-dependent manner [58].

### p53 and cancer

The p53 protein is a transcription factor located at the center of a large signaling network. It regulates expression of over 150 genes, including *p21*, *GADD45*, *MDM2*, *IGFBP3*, and *BAX *[70]. Some of the genes induced or inhibited by p53 are involved in such cellular processes as cell cycle progression, apoptosis induction, DNA repair, response to cellular stress, among other functions [71]. When p53 function is lost, either directly through mutation or indirectly through several other mechanisms, the cell often undergoes cancerous transformation [72]. For this reason, a loss of p53 function is considered as a major factor in cancer development [72].

To carry out its numerous signal transduction functions, p53 interacts physically with a large number of other proteins. Many of these interactors are transcription factors, and many more are activators or inhibitors of p53 transactivation activities. The p53-Mdm2 interaction is of special interest due to its direct relation to the oncogenesis. The Mdm2 protein inactivates p53 by binding to its transcription activation domain [73]. This interaction prevents p53 from activating its target genes in three ways [74]: (*i*) It directly blocks p53 from binding to various transcription factors; (*ii*) Mdm2 acts as a ubiquitin ligase, targeting p53 for destruction; (*iii*) Mdm2 contains a nuclear export signal, so the p53-Mdm2 complex tends to be exported from the nucleus, thereby preventing p53 from activating genes.

**Figure 3:**
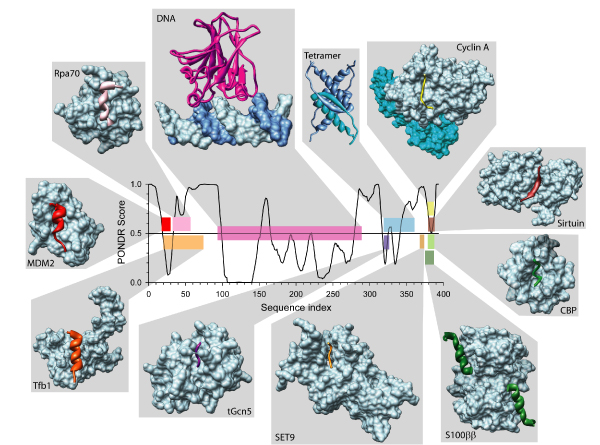
**Disorder profile and functionality of p53**. Intrinsic disorder was predicted by the PONDR^® ^VLXT. Segments with scores above 0.5 correspond to disordered regions, while those below 0.5 correspond to ordered regions/binding sites. p53 is at the center of a large signaling network, regulating expression of genes involved in a variety of cellular processes and interacting with a large number of other proteins. The interaction sites are signaled by downward spikes in the plot of the predicted disorder. The structures of the complexes containing various p53 binding regions are displayed around the predicted disorder pattern. In complexes, the structures of p53 segments bound to their partners are shown in different colors. These color codes are also used for bars in the PONDR^® ^VLXT plot to indicate the positions of the regions of known structure in the context of the intrinsic disorder predictions. The Protein Data Bank IDs and partner names for the structures (from upper left, clockwise) are as follows: (1tsr DNA), (1gzh 53BP1), (1q2d gcn5), (3sak p53 (tet dom)), (1xqh set9), (1h26 cyclinA), (1ma3 sirtuin), (1jsp CBP bromo domain), (1dt7 s100bb), (2h1l sv40 Large T antigen), (1ycs 53BP2), (2gs0 PH), (1ycr MDM2), and (2b3g rpa70).

Several interactions have been mapped to the N-terminal domain (i.e., the transactivation domain), the C-terminal domain (i.e., the regulatory domain), and the DNA binding domain (DBD) of p53 [58,71]. These domains have also been characterized in terms of their intrinsic disorder-order state, where the DNA binding domain is intrinsically structured and the terminal domains are intrinsically disordered [51,58,75,76]. Additionally, many sites of various posttranslational modifications have been identified in p53. Overall, ~70% of the interactions are mediated by IDRs in p53 [51]. A bias toward intrinsic disorder is even more pronounced in the sites of posttranslational modifications, with 86%, 90%, and 100% of observed acetylation, phosphorylation, and protein conjugation sites, respectively, found in IDRs [51,58]. Clearly, p53 extensively uses disordered regions to mediate and modulate interactions with other proteins. This is illustrated by Figure 3, which represents a set of complexes of various p53 fragments or domains with numerous binding partners.

### Intrinsic disorder, HPV proteins and cervical cancer

There are more than 100 different types of human papillomaviruses (HPVs), which are the causative agents of benign papillomas/warts, and cofactors in the development of carcinomas of the genital tract, head and neck and epidermis. With respect to their association with cancer, HPVs are grouped into two classes, known as low- (e.g., HPV-6 and HPV-11) and high-risk (e.g., HPV-16 and HPV-18) types. The entire proteome of HPV includes two structural proteins, L1 and L2, and six nonstructural proteins: E1, E2, E4, E5, E6 and E7. The last two, E6 and E7, are known to function as oncoproteins in the high-risk HPVs. The correlation between the amount of ID and the ability of human papillomaviruses to cause the carcinoma development has been recently evaluated [77]. To this end, a detailed bioinformatics analysis of proteomes of high-risk and low-risk HPVs with the major focus on E6 and E7 oncoproteins was performed. The results of this analysis were consistent with the conclusion that high-risk HPVs were characterized by the increased amount of intrinsic disorder in transforming proteins E6 and E7 [77].

## IDPs in human diseases: from individual cases to general picture

Although the illustrative examples given above demonstrate the involvement of IDPs and IDRs in various diseases, and despite several more cases that are scattered in literature, all of these examples together are not sufficient to determine the extent of IDP involvement in the pathogenesis of human diseases. Simply put, how generally do IDPs and IDRs play important roles in human disease? To answer this big question, appropriate analytical tools are needed. In a fashion similar to the history of the development of the IDP concept itself, bioinformatics is being used to determine the extent and generality of the involvement of IDPs and IDRs in human disease.

### Unfoldomics of human diseases: tools to establish and analyze disease-related unfoldome

#### Unfoldome and unfoldomics

Since IDPs are highly abundant in various diseases (see below), the "disorder in disorders" or D^2 ^concept was introduced to summarize work in this area [58]. As the number of IDPs related to various diseases is very large, it makes sense to develop the disease-related unfoldome and unfoldomics concepts.

The use of the suffix '-ome' has a long history while '-omics' is much more recent. The Oxford English Dictionary (OED) attributes 'genome' to Hans Winkler from his 1920 work [78]. While the OED suggests that 'genome' arose as a portmanteau of 'gene' and 'chromosome', this does not seem to be supported by the literature. Instead, Lederberg and McCray suggest that, as a botanist, Winkler must have been familiar with terms such as biome (a biological community), rhizome (a root system), and phyllome (the leaves covering a tree) among others, all of which were in use well before 1920 and all of which signify the collectivity of the units involved [79]. Thus, 'ome' implies the complete set of the objects in question, with genome signifying the set of genes of an organism. By changing the 'e' in '-ome' to '-ics', the new word is created that indicates the scientific study of the '-ome' in question. For genome, the change to 'genomics' did not occur until 1987 when a journal by this name was founded by Victor McKusick and Frank Ruddle [79].

Many additional conversions from -ome to -omics have subsequently occurred and a large number of "-omes" have been accepted in biology, including but not limited to the following: genome, proteome, interactome, metabolome, transcriptome, diseasome, toxicogenome, nutrigenome, cytome, oncoproteome, epitome, and glycome, etc. For a more complete list, the reader is directed to . Interestingly, some of the -ome words at this website cannot be found in PubMed searches, whereas similar words can be found. For example, 'foldome' and 'foldomics' are both listed on this website, but a search of these words in PubMed yields no hits for either word, while the similar word, 'foldeomics,' yields one hit, which leads to a database containing information about protein folding. The suffixes -ome and -omics imply a new layer of knowledge, especially when a scientist is dealing with the data produced by the large-scale studies, including the high throughput experiments and the computational/bioinformatics analyses of the large datasets.

The unfoldome and unfoldomics concepts are built on the ideas given above. Unfoldomics is the field that focuses on the unfoldome. The unfoldome is the set of IDPs, which are also known as natively unfolded proteins, hence the unfoldome. We are also using unfoldome to cover segments or regions of proteins that remain unfolded in the functional state. Unfoldomics considers not only the identities of the set of proteins and protein regions in the unfoldome of a given organism, but also their functions, structures, interactions, evolution, etc. Because IDPs and IDRs are highly abundant in nature (~50% eukaryotic proteins are either entirely disordered or contain long disordered regions), have amazing structural variability and possess a very wide variety of functions, we thought it appropriate to name this realm of proteins the unfoldome, with unfoldomics reflecting the totality of the phenomena associated with IDPs and IDRs.

#### Computational tools for the unfoldome analysis

Obviously, when the scale of analysis increases from one protein to many, new analytical tools are required. The set of computational tools utilized in the bioinformatics studies on disease-related unfoldomes is briefly introduced below. This set includes compositional profiling, disorder prediction, evaluation of the number of potential binding sites, analysis of alternative splicing, and determination of posttranslational modifications.

##### Compositional profiling

A specific feature of a probable IDR is its amino-acid compositional bias characterized by a low content of so-called order-promoting residues such as Cys, Trp, Phe, Tyr, Val, Leu, and Ile, and a high content of so-called disorder-promoting residues, Glu, Lys, Arg, Asp, Gln, Ser, Pro, and Thr [7,45,80,81]. This bias can be visualized by plotting the fractional difference in composition between a given set of proteins and a set of ordered proteins [7,81]. These fractional differences in composition between the studied set and a set of ordered proteins are calculated for each amino acid residue as (C_x_-C_order_)/C_order_, where C_x _is the content of a given amino acid in the set of interest, and C_order _is the corresponding content in a set of ordered proteins. The analysis can be performed using a web Composition Profiler tool .

##### Disorder predictions

Predictions of the intrinsic disorder propensity can be performed using a set of per-residue Predictors Of Natural disordered regions (PONDR^®^) algorithms, PONDR^® ^VLXT, VL3 and VSL1/2 or a set of binary predictors that predict disorder on the level of whole proteins, charge-hydrophathy plot (CH-plot) and cumulative distribution function (CDF) analysis. Many research groups have developed a number of different predictors of disorder in addition to the examples listed above. Links to many of these predictors can be found at .

PONDR^® ^VLXT combines three neural networks, one for internal sequences and one for each terminus of the sequence. The internal predictor was trained on disordered sequences from only 15 proteins whose disorder was characterized by either X-ray or NMR studies [80]. The terminal predictors were trained on short regions of X-ray characterized disorder from the N- and C-terminus [82]. The merger was accomplished by performing overlapping predictions, followed by averaging the outputs. The VLXT training set included disordered segments of 40 or more amino acid residues as characterized by X-ray and NMR for the predictor of the internal regions, and segments of five or more amino acid residues for the predictors of the two terminal regions. VLXT most likely underestimates the occurrence of long disordered regions in proteins. However, this algorithm is very important for finding potential binding sites (see below).

PONDR^® ^VL3 combines the predictions of 30 neural networks for the entire protein sequence and was trained using disordered regions from more than 150 proteins characterized by the methods mentioned above plus circular dichroism, limited proteolysis and other physical approaches [83]. This is one of the most accurate predictors of long disordered regions.

PONDR^® ^VSL1/2 is a recently developed Various Short-Long, version 1/2 (PONDR^® ^VSL1/2) algorithm, which is an ensemble of logistic regression models that predict per-residue order-disorder [84,85]. Two models predict either long or short disordered regions – greater or less than 30 residues – based on features similar to those used by VLXT. The algorithm calculates a weighted average of these predictions, where the weights are determined by a meta-predictor that approximates the likelihood of a long disordered region within its 61-residue window. Predictor inputs include PSI-BLAST [86] profiles and PHD [87], and PSI-PRED secondary structure predictions [88].

##### CDF analysis

Originally, cumulative distribution function (CDF) analysis summarized the per-residue disorder predictions by plotting PONDR^® ^VLXT scores [80,82,89] against their cumulative frequency, which allows ordered and disordered proteins to be distinguished based on the distribution of prediction scores [47]. At any given point on the CDF curve, the ordinate gives the proportion of residues with a PONDR^® ^score less than or equal to the abscissa. The optimal boundary that provided the most accurate order-disorder classification was shown to represent seven points located in the 12^*th *^through 18^*th *^bin [47]. Thus, for CDF analysis, order-disorder classification is based on whether a CDF curve is above or below a majority of boundary points. Recently, CDF analysis was extended to include several other per-residue predictors of intrinsic disorder [90].

##### CH-plot analysis

Ordered and intrinsically unstructured proteins occupy non-overlapping regions in the charge-hydropathy plots (CH-plots), with natively unfolded proteins being specifically localized within a particular region of charge-hydropathy phase space, satisfying the following relationship [6,47]:



where ⟨*H*⟩ and ⟨*R*⟩ are the mean hydropathy and the mean net charge of the given protein, respectively, whereas ⟨*H*⟩_*b *_is the "boundary" mean hydropathy value, below which a polypeptide chain with a given ⟨*R*⟩ will be most probably unfolded. The mean hydropathy, ⟨*H*⟩, is defined as the sum of the normalized hydropathy of all residues divided by the number of residues in the polypeptide. The mean net charge ⟨*R*⟩ is defined as the net charge at pH 7.0, divided by the total number of residues [6,47].

##### α-MoRF predicitions

The order/disorder tendencies of IDPs as revealed by PONDR^® ^VLXT could be used to find disordered region(s) involved in interaction with specific binding partners. In fact, often IDPs have a peculiar and well-recognizable pattern, where short region of predicted order is surrounded by extended regions predicted disorder. This specific pattern was used to develop a unique bioinformatics tool dedicated to the identification of potential protein-protein interaction sites in IDPs, namely the identifier of α-helix forming Molecular Recognition Features, α-MoRF, which is focused on short binding regions within long regions of disorder that are likely to form helical structure upon binding [19,91]. The predictor utilizes a stacked architecture, where PONDR^® ^VLXT is used to identify short predictions of order within long predictions of disorder and then a second level predictor determines whether the order prediction is likely to be a binding site based on attributes of both the predicted ordered region and the predicted surrounding disordered region. An α-MoRF prediction indicates the presence of a relatively short (20 residues), loosely structured helical region within a largely disordered sequence [19,91]. Such regions gain functionality upon a disorder-to-order transition induced by binding to partner.

##### Alternative splicing analysis

Alternative splicing (AS) is a process responsible for the production of multiple, mature mRNAs from a single precursor pre-mRNA by the inclusion and omission of different segments [92]. Therefore, the AS regions are defined as exons or parts of exons that are expressed in some, but not in all protein sequences transcribed from a given gene. AS is prevalent in multicellular eukaryotes [93], and it is estimated that 40 – 60% of human genes yield multiple proteins via this process [94]. These observations suggest that AS provides an important mechanism for enhancing the diversity of the proteome in multicellular eukaryotes [95]. As AS impacts many protein functions such as ligand binding, enzymatic activity, and protein-protein interactions, not surprisingly, abnormal AS has been associated with various human diseases, including myotonic dystrophy [96], axoospermia [97], Alzheimer's disease [98], cancer [99,100] and many others.

In the disease-related unfoldome, the sequence alignments of genes with multiple isoforms provide information on the AS regions. Similarly as for a whole protein, disorder content for an AS region is estimated as the fraction of its residues that are predicted to be disordered.

##### PTM analysis

Posttranslational modifications (PTMs) are widely employed by cells to modulate the functionalities of many of their proteins. Some proteins require different types of posttranslational modifications for their function. PTMs are classified according to the mechanisms that are involved: addition of functional groups (e.g., acylation, alkylation, phosphorylation, glucosylation, etc.); attachement of other proteins and peptides (e.g., ubiquitination, SUMOylation, etc.); changing of the chemical nature of amino acids (deamidation, deimidation, oxidation, etc.); and dissection of the backbone by proteolytic cleavage. Additionally, according to the conformational state of the potential PTM site, PTMs can be grouped into two major classes. The first class involves modifications that are associated primarily with structured proteins and regions, whereas the second class combines modifications that are associated primarily with IDPs and IDRs [50]. The first class of PTMs is crucial for providing moieties for catalytic functions, for modifying enzyme activities or for stabilizing protein structure. This includes formylation, protein splicing, oxidation and covalent attachment of quinones and organic radicals [50]. The abundance of IDRs among the primary targets for the second class PTMs is likely determined by the need of the modifying enzymes to bind to their corresponding substrates via high specificity/low affinity interactions; such characteristics are typical of signaling interactions and typically involve disorder-to-order transitions of at least one of the partners [50]. Among the second class of PTMs are phosphorylation, acetylation, acylation, adenylylation, ADP ribosylation, amidation, carboxylation, formylation, glycosylation, methylation, sulfation, prenylation, ubiquitination, and Ubl-conjugation (i.e., covalent attachment of ubiquitin-like proteins, including SUMO, ISG15, Nedd8, and Atg8) [50].

As amino acid compositions, sequence complexity, hydrophobicity, charge and other sequence attributes of regions adjacent to phosphorylation sites were found to be very similar to those of IDPs and IDRs, a specific web-based tool for the prediction of protein phosphorylation sites, DISPHOS (DISorder-enhanced PHOSphorylation predictor, ) was elaborated [101]. Recent studies further support the view that phosphorylation occurs much more often in IDPs and IDRs as compared to structured proteins and regions [102,103]. A predictive tool similar to DISPHOS is also available for protein methylation [104]. These tools can be utilized to evaluate the abundance of PTMs in the disease-related unfoldome.

#### Establishing and analyzing the disease-related unfoldomes

Three approaches were elaborated to estimate the abundance of IDPs in various pathological conditions. The first approach is based on the assembly of specific datasets of proteins associated with a given disease and the computational analysis of these datasets using a number of disorder predictors [9,58,77,105]. In essence, this is an analysis of individual proteins extended to a set of independent proteins. A second approach utilized network of genetic diseases where the related proteins are interlinked within one disease and between different diseases [106]. A third approach is based on the evaluation of the association between a particular protein function (including the disease-specific functional keywords) with the level of intrinsic disorder in a set of proteins known to carry out this function [48-50]. These three approaches are briefly described below, whereas the results of their application are presented in the subsequent section.

##### Simple dataset analysis

The simplest analysis of the abundance of intrinsic disorder in a given disease is based on the two-stage protocol, where a set of related proteins is first assembled by searching various databases and then the collected group of proteins is analyzed for intrinsic disorder. The depth of this analysis is based on the breadth of the search for the disease-related proteins and on the number of different computational tools utilized to find disordered proteins/regions. For example, a dataset of human cancer-associated proteins (HCAP) extracted from SWISS-PROT  using keywords Anti-oncogene; Oncogene; Proto-oncogene; tumor in the description field and "human" in the organism field contained 231 proteins [9]. Whereas 487 proteins associated with cardiovascular disease (CVD) were found in SWISS-PROT using an exhaustive list of CVD-related keywords: Aneurysm; Angina Pectoris; Angioneurotic Edema; Aortic Valve Stenosis; Arrhythmia; Arrhythmogenic; Arteriosclerosis; Arteriovenous Malformations; Atrial Fibrillation; Behcet Syndrome; Bradycardia; Cardiac Tamponade; Cardiomegaly; Cardiomyopathy; Cardiovascular Disease; Carotid Stenosis; Cerebral Hemorrhage; Churg-Strauss Syndrome; Ebstein's Anomaly; Eisenmenger Complex; Embolism; Cholesterol; Endocarditis; Fibromuscular Dysplasia; Heart Block; Heart Defects; Heart Disease; Heart Failure; Heart Valve Diseases; Hematoma; Hippel Lindau Disease; Hyperemia; Hypertension; Hypertrophy; Hypoplastic Left Heart Syndrome; Hypotension; Intermittent Claudication; Klippel-Trenaunay-Weber Syndrome; Lateral Medullary Syndrome; Long QT Syndrome; Microvascular AnginaOR Mitral Valve Prolapse; Moyamoya Disease; Mucocutaneous Lymph Node Syndrome; Myocardial Infarction; Myocardial Ischemia; Myocarditis; Pericarditis; Peripheral Vascular Diseases; Phlebitis; Polyarteritis Nodosa; Pulmonary Atresia; Raynaud Disease; Sneddon Syndrome; Superior Vena Cava Syndrome; Tachycardia; Takayasu's Arteritis; Telangiectasia; Telangiectasis; Temporal Arteritis; Tetralogy of Fallot; Thromboangiitis Obliterans; Thrombosis; Tricuspid Atresia; Varicose Veins; Vascular Disease; Vasculitis; Vasospasm; Ventricular Fibrillation; Williams Syndrome; Wolff-Parkinson-White Syndrome; Heart disease; Stroke; Thromb; Cardio-vascular disease; Blood coagulation; Heart muscle; Cardiovascular disease; Plasma; Vascular disease in the description field and "human" in the organism field [105]. The intrinsic disorder analysis in the assembled datasets of disease-related proteins includes various computational tools described in a previous section.

##### Functional keyword analysis

A computational tool for the evaluation of a correlation between the functional annotations in the SWISSPROT database and the predicted intrinsic disorder was elaborated [48-50]. First, functional keywords associated with 20 or more proteins in SWISSPROT were determined and corresponding protein datasets were assembled. Then, for each keyword-associated set, a length-matching set of random proteins was drawn from the SWISSPROT. Order-disorder predictions were carried out for the keyword-associated sets and for the random sets. If a function described by a given keyword were carried out by a long region of disordered protein, one would expect the keyword-associated set to have a greater amount of predicted disorder compared to the random set. The keyword-associated set would have less prediction of disorder compared to the random set if the keyword-associated function were carried out by structured protein. Given the two sets of predictions for the pairs of sets, it is possible to calculate the p-values, where a p-value > 0.95 suggests a disorder-associated function, a p-value < 0.05 suggests an order-associated function, and intermediate p-values are ambiguous [48-50].

##### Genetic diseasenNetwork analysis

To estimate whether human genetic diseases and the corresponding disease genes are related to each other at a higher level of cellular and organism organization, a bipartite graph was utilized in a dual way: to represent a network of genetic diseases, the "human disease network", HDN, where two diseases are directly linked if there is a gene that is directly related to both of them, and a network of disease genes, the "disease gene network", DGN, where two genes are directly linked if there is a disease to which they are both directly related [107]. This framework, called the human diseasome, systematically linked the human disease phenome (which includes all the human genetic diseases) with the human disease genome (which contains all the disease-related genes). This diseasome opened a new avenue for the analysis and understanding of human genetic diseases, moving from single gene-single disease viewpoint to a framework-based approach [107].

Using this approach various diseases were classified into 20 types, some diseases were unclassified, and several diseases were annotated as belonging to multiple classes. Similarly, genes were clustered into classes via their associations with specific diseases [107]. The analysis of these networks revealed that of 1,284 genetic diseases, 867 had at least one link to other diseases, and 516 diseases formed a giant component, suggesting that the genetic origins of most diseases, to some extent, were shared with other diseases. Similalrly, in the DGN, 1,377 of 1,777 disease genes were shown to be connected to other disease genes, and 903 genes belonged to a giant cluster [107]. The vast majority of genes associated with genetic diseases was non-essential and showed no tendency to encode hub proteins. In fact, many of the disease-related genes were shown to be localized in the functional periphery of the network [107]. The large-scale analysis of the abundance of intrinsic disorder in transcripts of the various disease-related genes was performed using a set of computational tools described in a previous section [106]. The results of this analysis suggest that IDPs are broadly involved in human diseases (see below).

### IDPs in cancer, CVD, neurodegenerative diseases and diabetes

**Figure 4:**
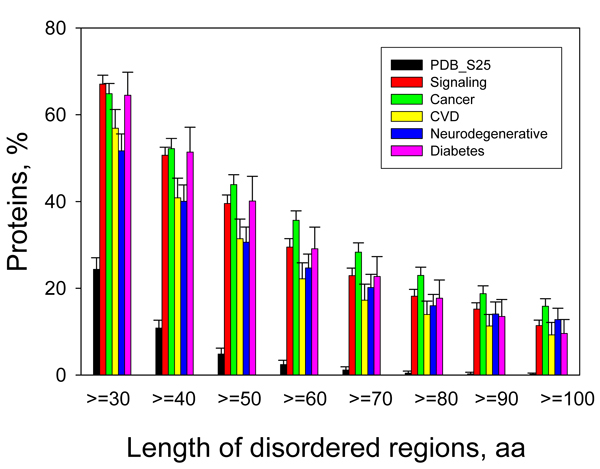
**Abundance of intrinsic disorder in disease-associated proteins**. Percentages of disease associated proteins with ≥ 30 to ≥ 100 consecutive residues predicted to be disordered. The error bars represent 95% confidence intervals and were calculated using 1,000 bootstrap re-sampling. Corresponding data for signaling and ordered proteins are shown for the comparison. Analyzed sets of disease-related proteins included 1786, 487, 689, and 285 proteins for cancer, CVD, neurodegenerative disease and diabetes, respectively.

For the first time, the dataset analysis approach was used in 2002 [9], when significant fractions of cancer-associated and cell-signaling proteins were found to contain predicted IDRs of 30 residues or longer (see Figure 4). This was in a sharp contrast to a set of structured (ordered) proteins with well-defined 3-D structures, which was shown to contain only 13% of the proteins with predicted IDRs ≥ 30 residues. Following a similar analytical model, a dataset of 487 proteins related to cardiovascular disease (CVD) was collected and analyzed [105]. On average, CVD-related proteins were found to be highly disordered. They were depleted in major order-promoting residues (Trp, Phe, Tyr, Ile, and Val) and enriched in some disorder-promoting residues (Arg, Gln, Ser, Pro, and Glu). High level of intrinsic disorder and a substantial number of potential interaction sites were also found using a set of computational tools. The percentage of proteins with 30 or more consecutive disordered residues was ~60% for CVD-associated proteins (see Figure 4). Many proteins were predicted to be wholly disordered, with 101 proteins from the CVD dataset predicted to have a total of almost 200 specific disorder-based binding motifs (thus about 2 binding sites per protein). These binding sites are called α-helical molecular recognition features, α-MoRFs, and have been well studied from protein complexes taken from PDB [105]. All of this clearly suggested that IDPs might play key roles in CVD.

In addition to being abundant in cancer- and CVD-related proteins, intrinsic disorder was commonly found in such maladies as neurodegenerative diseases and diabetes. Figure 4 represents this as the percentages of proteins with ≥ 30 consecutive residues predicted to be disordered in datasets of proteins associated with all four diseases. This figure shows that intrinsic disorder is highly prevalent in proteins associated with all of the studied diseases, being comparable with that of signaling proteins and significantly exceeding the levels of intrinsic disorder in eukaryotic and in non-homologous, structured proteins [58].

### Functional anthology of intrinsic disorder and human diseases

The application of the functional keyword analysis tool revealed that out of 710 SWISSPROT keywords each being assigned to at least 20 proteins, 310 had p-values < 0.05, suggesting order-associated functions, 238 had p-values > 0.95, suggesting disorder-associated functions, and the remainder, 162, gave intermediate p-values, yielding ambiguity in the likely function-structure associations [48-50].

**Figure 5:**
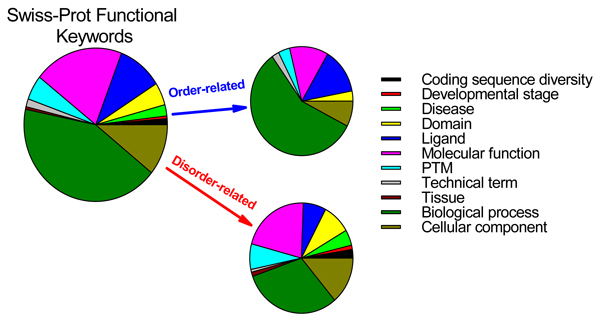
**Functional anthology of IDPs**. More than 200,000 proteins and 710 SWISSPROT functional keywords each associated with at least 20 different proteins were analyzed [48-50]. Based on the bioinformatics analysis, 238 keywords were associated with the predicted intrinsic disorder. These keywords covered various functions and included almost all disease-related keywords. This is in a strict contrast to 302 keywords which were associated with the predicted order. Functionally, the vast majority of these keywords were various "ases". They contained almost no disease-related keywords.

When the functional keywords were partitioned into eleven functional categories (Biological processes, cellular components, developmental stage, etc.) order-associated keywords were found for seven of the categories, but disorder-associated keywords were found for all eleven categories [48]. Figure 5 represents the results of this analysis and show that many diseases were strongly correlated with proteins predicted to be disordered. Contrary to this, we did not find disease-associated proteins to be strongly correlated with absence of disorder [50]. Among disease-related Swiss-Prot keywords strongly associated with intrinsic disorder were oncoproteins, malaria, trypanosomiasis, human immunodeficiency virus (HIV) and acquired immunodeficiency syndrome (AIDS), deafness, obesity, cardiovascular disease, diabetes mellitus, albinism, and prion [50]. In agreement with this bioinformatics analysis, we were able to find at least one illustrative, experimentally validated example of functional disorder or order for the vast majority of functional keywords related to diseases [50].

### Intrinsic disorder in proteins from the genetic disease network

The dual Human Disease Network/Disease Gene Network (HDN/DGN) consists of two types of nodes that represent human genes (1,777) and diseases (1,284), and links that connect diseases with related genes [107]. A set of disease genes from DGN with human genes with known protein sequences was used to collect protein sequences for all human genes from NCBI Gene database [106]. All model proteins obtained solely with automated genome annotation processing were excluded from the consideration. After this exclusion, the diseasome included 1,751 human disease related genes. The transcripts of the genetic disease-associated genes were compared with proteins encoded by 16,358 other human genes with known protein sequences [106].

The abundance of intrinsic disorder in these diseasome network proteins was evaluated by means of several prediction algorithms, including PONDR^® ^VSL2, CDF-analysis, CH-plot [106]. The functional repertoires of these proteins were analyzed based on prior studies relating disorder to function [48-50]. These analyses uncovered an unfoldome associated with human genetic diseases and revealed several interesting peculiarities [106]:

(i) Intrinsic disorder is common in proteins associated with many human genetic diseases;

(ii) Different disease classes vary significantly in the IDP contents of their associated proteins;

(iii) Molecular recognition features, which are relatively short loosely structured protein regions within mostly disordered sequences and which gain structure upon binding to partners, are common in the diseasome, and their abundance correlates with the intrinsic disorder level;

(iv) Some disease classes have a significantly higher fraction of genes affected by alternative splicing, and the alternatively spliced regions in the corresponding proteins are predicted to be highly disordered and in some disease classes contain a significantly higher number of MoRFs;

(v) Correlations were found among the various diseasome graph-related properties and intrinsic disorder. In agreement with earlier studies, hub proteins were shown to be more disordered.

### Why the unfoldome and why IDPs?

All the data presented above provide evidence that IDPs are very common in various diseases and therefore comprise a disease-related unfoldome. The introduction of the unfoldome and unfoldomics concepts pave the way for a better understanding of the molecular aspects of human diseases, including a better understanding of their pathogenesis and molecular mechanisms. This concept is also important for the development of the appropriate strategies dedicated to the targeted analysis of the disease-related proteins. As many of these proteins are either completely disordered or contain long disordered regions, it would be a clear mistake to analyze them using only the experimental tools developed for the characterization of structured proteins. The appropriate conformational analyses should utilize the fact that IDPs and IDRs possess a range of structural properties that are quite different from those of ordered proteins [6,7,11,12,14,21]. The techniques used for such analysis were described in a recent review [57]. Some of these techniques are briefly considered below.

(i) Although X-ray crystallography is traditionally used to characterize structure of ordered proteins, it repeatedly defines missing electron density in many protein structures, which may correspond to disordered region(s). The increased flexibility of atoms in the IDR leads to the non-coherent X-ray scattering, making them unobserved or at least smearing out their electron densities. Missing regions of structure can be structured but wobbly domains rather than disordered regions, and so further studies on X-ray identified IDRs using other methods is very important.

(ii) A solution-based counterpart of X-ray crystallography is heteronuclear multidimensional NMR. This is an extremely powerful technique for protein 3D-structure determination in solution and for the characterization of protein dynamics. Recent advances in this technology have allowed the complete assignment of resonances for several unfolded and partially folded proteins, as well as for the several IDPs and IDRs.

(iii) Circular dichroism (CD) is another powerful technique for the evaluation of the overall tertiary structure of a protein. CD spectra in the near UV region (250–350 nm) reflect the asymmetry of the environment of aromatic amino acid residues and, consequently, are characteristic of protein tertiary structure. IDPs may be detected by their display of simplified near-UV CD spectra.

(iv) Decreased content of ordered secondary structure in IDPs may be detected by several spectroscopic techniques including far-UV CD, optical rotary dispersion (ORD), Fourier transform infrared spectroscopy (FTIR), Raman optical activity and deep UV Raman spectroscopy.

(v) Hydrodynamic parameters obtained from techniques such as gel-filtration, viscometry, small angle X-ray or neutron scattering (SAXS or SANS, respectively), sedimentation, dynamic and static light scattering may help in determining the degree of a polypeptide chain compaction.

(vi) Another very important structural parameter is the degree of globularity, which reflects the presence or absence of a tightly packed core in a protein molecule. This information may be extracted from the analysis of SAXS data in form of a Kratky plot, the shape of which is sensitive to the conformational state of the scattering protein molecules. The Kratky plot of a globular molecule (ordered or molten globular) has a characteristic maximum, which is absent from the Kratky plot of a coil-like or pre-molten globule-like IDP.

(vii) Different fluorescence characteristics provide a wealth of knowledge on the intramolecular mobility and compactness of a protein. This includes FRET, shape and position of the intrinsic fluorescence spectrum, fluorescence anisotropy and lifetime, accessibility of the chromophore groups to external quenchers, and steady state and time-resolved parameters of the fluorescent dyes.

(viii) Increased proteolytic degradation *in vitro *of IDPs and IDRs indirectly confirms their increased flexibility.

(ix) Protein disorder may also be evaluated by immunochemical methods or via the interaction with molecular chaperones.

(x) Finally, IDPs may be detected by their response to the environmental changes or via the analysis of protein conformational stability.

(xi) Aberrant mobility during the SDS-PAGE gel electrophoresis may be suggestive of intrinsic disorder since disordered proteins usually migrate slower than their calculated molecular weight.

As discussed above, IDPs and IDRs can be characterized by a variety of biophysical and biochemical methods. As a result, a very large number of disease-associated proteins have been experimentally shown to be IDPs or to contain IDRs as indicate by the illustrative examples at the beginning of this article. This leads naturally to the following question: from a biological perspective, why have such proteins been so heavily linked to human diseases? To answer this question, some specific features of IDPs that potentially make them key players in the development of pathological conditions need to be considered. Many of these features are linked to the function of IDPs in signaling, regulation and control. The list of these features includes [24]:

(i) Decoupled specificity and strength of binding leading to high-specificity-low-affinity interactions;

(ii) Increased speed of interaction due to greater capture radius and the ability to spatially search interaction space;

(iii) Flexible encounter complexes (less stringent spatial orientation requirements);

(iv) Controlled regulation via high sensitivity to proteolytic degradation when in the free state;

(v) Increased interaction (surface) area per residue;

(vi) A one-to-many binding mode and binding promiscuity by which a single IDP/IDR binds to multiple structurally diverse partners. This is accomplished by plasticity, by which a given IDR folds into distinctive conformations to accommodate the diverse binding sites of its different partners

(vii) A many-to-one binding mode, by which many different IDPs/IDRs bind to one site on a single ordered partner. Again this is accomplished by plasticity, by which different IDRs fold into similar conformations that all fit into a single binding site on one partner.

(viii) Induced folding where an IDR folds as it binds to a specific partner;

(ix) Low steric restrictions allowing the elongation or contraction of a given binding area;

(x) Ease of regulation or reorganization of signaling networks by posttranslational modification;

(xi) Ease of regulation or reorganization of signaling networks by alternative splicing;

(xii) Overlapping of binding sites due to use of extended linear conformations for association;

(xiii) High evolutionary rates leading to rapid adaptability and easy modification of signaling networks;

(xiv) Flexibility that allows masking (or not) of interaction sites or that allows multiple interactions between bound partners.

### Induced folding, binding promiscuity, and binding plasticity

Protein-protein and protein-nucleic acid interactions are central to many processes in molecular biology. They often involve coupled folding and binding of at least one of the partners [4,6-8,10,13,17,22,108,109]. Among the list of structural features that make IDPs especially useful for their signaling and regulation functions include induced folding, binding promiscuity, and binding plasticity. The p53 protein molecule represents an especially dramatic example for which intrinsic disorder is heavily utilized for function via induced folding, binding promiscuity (i.e., the ability of a given IDP to bind interact with several binding partners), and binding plasticity (which is determined as the ability of a given IDR to gain different folds to accommodate diverse binding sites of different partners). As it has been already mentioned, p53 regulates expression of over 150 genes and binds to over 100 proteins [51,70,71]. These many interactions represent an illustrative example of the one-to-many biding mode [51]. The 3-D structures of several complexes between the various p53 regions and unique binding partners have been determined (see Figure 3). The interactions with 10 of these partners are mediated by region experimentally characterized as IDRs. Figure 3 shows that PONDR^® ^VLXT is able to detect the majority of these binding regions as short predictions of order within a longer prediction of disorder. These structures are complexes between p53 and: cyclin A, sirtuin, CBP, S100ββ, set9, tGcn5, Rpa70, Mdm2, Tfb1, and itself. The remaining 4 interactions are mediated by the structured DBD, between p53 and: DNA, 53BP1, sv40 Large T antigen, and 53BP2 [51].

Of special interest is the C-terminal regulatory domain, which is involved in the formation of multiple complexes. Figure 3 shows that a single IDR of p53 derived from the C-terminal regulatory domain (residues 374–388) was observed to form all three major secondary structure types in the bound state: a helix when associating with S100ββ, a sheet with sirtuin, an irregular structure with CBP, and an irregular structure with a completely different trajectory with cyclin A2. The set of residues involved in these interactions exhibit a very high extent of overlap along the sequence [51]. Based on the fact that the secondary structures adopted by this IDR in different complexes were very distinct, it seemed reasonable to expect that p53 utilizes different residues for the interactions with these four different binding partners. This hypothesis is supported via the quantification of the buried surface area for each residue in each interaction by calculating their ΔASA [51]. In fact, the ΔASA-based binding profiles for the single IDR of p53 bound to four different partners were completely different, indicating that the same residues were used to different extents in the four interfaces, suggesting that the same IDR sequence is "read" by the different partners in entirely different ways [51].

This intriguing p53 example demonstrates the roles of IDRs in determining multiple specificities associated with the one-to-many binding mode, where remarkable conformational changes enable very distinct surfaces to be formed for binding to different partners. The mentioned interactions of the C-terminal regulatory domain of p53 with various binding partners are used for the activation or inhibition of its primary role as a transcription regulator. Therefore, it is possible that the disordered binding regions may play a passive regulatory role by providing a specific binding site, where IDRs serve as the identification sites of the protein to be regulated [13,51].

Because p53 is so heavily studied, we have learned about the use of IDPs and IDRs for its functions, especially as providing sites for protein-protein interactions, before we have gained such knowledge for other signaling proteins. However, sites of protein-protein interactions that are located within IDPs and IDRs and that are very similar to those observed for p53 are predicted to be extremely common in the proteins of mammalian proteomes [91]. Thus, what we have presented above for p53 likely provides a blueprint for the use of IDPs and IDRs for a very large number of proteins in the cell.

Overall, there are intriguing interconnections among intrinsic disorder, cell signaling and human diseases, suggesting that protein conformational diseases may result not only from protein misfolding, but also from misidentification and missignaling.

## Concluding remarks

Intrinsic disorder is highly abundant among proteins associated with various human diseases. This conclusion is based on the detailed analysis of several well-characterized disease-related IDPs and on the results of the extensive bioinformatics studies. As the number of disease-related IDPs is very large and as many of these proteins are interlinked, the concepts of the disease-related unfoldome and unfoldomics were introduced. Here, the disease-related unfoldome is attributed to a significant part of human proteome, which includes malady-associated IDPs, their functions, structures, interactions, evolution, etc. We believe that the unfoldomics concept helps lead to better understanding of various human diseases, their pathogenesis and molecular mechanisms. This concept might also help in the development of specialized strategies for the targeted analysis of functional and structural properties of disease-related proteins. The high degree of association between intrinsic disorder and many proteins implicated in various maladies is due to structural and functional peculiarities of IDPs and IDRs, which are typically involved in cellular regulation, recognition and signal transduction. One of the promising future developments in the field of the disease-related unfoldome and unfoldomics is the evaluation of IDP/IDR abundance in the framework of disease ontology. However, since the corresponding resources are not ready yet, such an analysis might be difficult at the current point.

## Competing interests

The authors declare that they have no competing interests.

## Authors' contributions

VNU was involved in design and planning of all the experiments, drafted the manuscript, revised the final version and headed the project. CJO, BX, UM, HX, SV and LMI performed the computational analysis, designed figures and contributed to the manuscript writing. ZO and AKD were involved in design and planning of all the experiments and contributed to the manuscript writing. All authors have read and approved the final manuscript.

## Supplementary Material

Additional file 1 Human neurodegenerative disorders characterized by the presence of the α-synuclein deposits. Click here for file
