# Supplementary material for: Unfoldomics of human diseases: linking protein intrinsic disorder with diseases
Source: BMC Genomics. 2009 Jul 7;10(Suppl 1):S7. doi: 10.1186/1471-2164-10-S1-S7 (PMC2709268; doi:10.1186/1471-2164-10-S1-S7)
Supplement: Additional file 1 — Human neurodegenerative disorders characterized by the presence of the α-synuclein deposits. [file 1471-2164-10-S1-S7-S1.doc]

***Diseases with neuronal inclusions***

Normal aging

Parkinson's disease

Idiopathic

Neurotoxicant-induced (incidental)

Familial

With α-synuclein point mutations

With α-synuclein gene triplication

With mutations in other proteins

Pure autonomic failure

Lewy body dysphagia

Parkinsonism plus syndromes

Sporadic

Progressive supranuclear palsy

Olivoponto cerebrellar atrophy (Shy-Dragger syndrome)

Cortical-basal ganglionic degeneration

Sporadic pallidal degeneration

Bilateral striatopallido dentate calcinosis

Parkinsonism with neuroacanthocytosis

Familial

Familial diffuse Lewy body disease

Familial dementia with swollen achromatic neurons and cortico-basal inclusion bodies

Frontotemporal dementia with parkinsonism linked to chromosome 17

Associated with psychiatric disturbances

Associated with respiratory disturbances

Associated with dystonia

Associated with myoclonus and seizures

Familial progressive supranuclear palsy

Alzheimer's disease

Sporadic

Familial with APP mutation

Familial with PS-1 mutation

Familial with other mutations

Familial British dementia

Lewy body diseases

Dementia with Lewy bodies

Pure form - transitional/limbic

Pure form - neocortical

Diffuse Lewy body disease

Common form

Pure form

Lewy body variant of Alzheimer’s disease

Incidental Lewy body disease

Lewy body dementia

Senile dementia of Lewy body type

Dementia associated with cortical Lewy bodies

Down’s syndrome

Amyotrophic lateral sclerosis-parkinsonsim/dementia complex of Guam

Neuroaxonal dystrophies

Neurodegeneration with brain iron accumulation, type I (Hallervorden-Spatz syndrome or adult neuroaxonal dystrophy)

Motor neuron disease

Amyotrophic lateral sclerosis

Familial

Sporadic

Tauopathies

Frontotemporal degeneration/dementia

Pick's disease

Post-encephalitic parkinsonism

Dementia pugilistica

Argyrophilic grain disease

Corticobasal degeneration

Prion diseases

Transmissible spongiform encephalopathies

Sporadic

Creutzfeldt-Jakob disease

Familial

Familial Creutzfeldt-Jakob disease

Gertsmann-Straussler-Scheinker syndrome

Infectious

Iatrogenic Creutzfeldt-Jakob disease

Variant Creutzfeldt-Jakob disease

Kuru

Fatal familial insomnia

Ataxia telangiectatica

Meige's syndrome

***Diseases with neuronaland glial inclusions***

Multiple system atrophy

Shy-Drager syndrome

Striatonigral degeneration (MSA-P)

Olivopontocerebellar atrophy (MSA-C)
